# The Mother in the Factory.—II

**Published:** 1898-04-02

**Authors:** 


### MODERN SOCIOLOGY.

### THE MOTHER IN THE FACTORY.—II.

It has often been thought that the infantile death-rate might be a measure of the injury done by the employment of married women in factories. Thus in Blackburn, where the percentage of married women workers is reckoned to be at least 47, the deaths of infants amount to 207 per 1,000; in Preston the estimated percentage of workers is 39, and the infant deaths 244 per 1,000; while coming down the scale to towns where only 1 per cent. of the married women go out to work, or where the unmarried workers are in a majority, the deaths of infants sink to about 170 per 1,000. But, coincident with this scale, Miss Collet notes that the number of domestic servants progresses inversely. Thus, in Blackburn the percentage of women servants is 5, in Preston, 6; while in Birkenhead and the towns like it, where the unmarried outnumber the married women workers, the percentage of servants is 12 per cent. of the whole female population. The difficulty of drawing a safe inference is therefore great. As Miss Collet says: "Both the real percentage of married and widowed women occupied, and the infant mortality in working-class districts, are concealed by the presence of a servant-keeping class. But in so far as this method of comparing towns with regard to employment of married women is a test, it lends little support to the theory that differences in the extent of employment of married women in towns are the main causes of the differences in the infant mortality rates. . . . One thing only is clear, that to find out the causes of high rates of infant mortality it will not do to compare town with town. Working-class districts with low rates must be compared with working-class districts with high rates, and districts with high rates must be compared with each other."

To this end Miss Collet compares, not whole towns, but registration sub-districts. And the results thus obtained are surprising. The Trinity district of Preston leads the way with an infant death-rate of 266 per 1,000, and a percentage of married women workers of 45. But in the Dale Street district of Liverpool the married women who are employed are only 17·7 per cent., and the infant mortality shows a rate of 225 per 1,000. In the St. George district of the same town we find only 14 per cent. of the married women set down as workers, yet the infant mortality rate is 200 per 1,000. But worse than all is the case of Sunderland. There, with only 6·5 of the married women at work, we find that infants die at the rate of 223 per 1,000. Other towns show similarly, though not equally, erratic results. It is, however, evident that the employment of the mothers will not wholly account for the deaths of the children. On the contrary, the inference which Miss Collet draws is obvious: "Whatever may be the dangers to infant life in a district where nearly half the married women are full-time workers in a factory, the children of industrious mothers are in a better position than the children of idle women of irregular habits."

It is satisfactory to find that, on the whole, the number of married women who are at work outside their own homes is decreasing. During the recent census decades there has been a steady increase of women workers between the ages of 15 and 25, and a decrease among those both older and younger. Thus, in 1851 the number of girls over 10 and under 15 who were at work was estimated at 2,148 per 10,000 living. In 1891 the proportion had fallen to 1,626. This, however, is 120 per 10,000 more than in 1881, the returns for which year showed a remarkable diminution from the numbers of the preceding census period, due doubtless to the combined action of the Education and Factory Acts. Up to that time there had been a small decrease, but at this period the proportion fell abruptly from 2,120 per 10,000 in 1871 to 1,506 in 1881. But between the ages of 15 and 25 the increase has always been steady. Thus, in 1851 the number occupied between those ages was 5,652 per 10,000; in 1861, 5,936; in 1871, 6,123; in 1881, 6,214; and in 1891, 6,336 per 10,000. Some of these women, no doubt, are married, but seeing that the age-period starts so young, it is probable that the majority are not. The next age-period covers 20 years of life, from 25 to 45, and we may fairly assume that a large number of those comprised in it are married. Within it there is more variation than in the previous list. The number returned as employed was, in 1851, a little over 3,000 per 10,000 living; in the next census period it was 3,168; in 1871 this came down to 3,155; and in 1881 to 2,900. 1891 saw an increase to 2,960 per 10,000; but as 1881 was the first year that those who had retired from various occupations were not set down as belonging to them, it is possible that the increase of the last ten years is more apparent than real. But it is probably true that more women of comparatively mature years are employed now than in 1881. In all the occupations which educated women have taken up to any extent, the age of the workers is, in almost every case, over 25. Nurses, journalists, high school teachers, are, more often than not, past that age. Moreover, work has become a fashion. Women of limited income who would in former days have struggled along miserably on it, now frankly take up some occupation. Perhaps they worked in the old time also; but it was under the rose and apologetically, and they probably did not confess to it on a census paper; while women similarly placed would now be just as anxious to claim the distinction of being workers.

A certain amount of unreliability thus comes in; and, as Miss Collet suggests, the proverbial untruthfulness of women regarding their age might modify the conclusions to be drawn from the figures. But under 25, it is possible that the misstatement would be on the side of pretending to be older rather than younger. A girl going out to service will state her age at her next instead of her last birthday to her employer, because the greater age gives a kind of claim to a bigger wage; and some of them make statements much wider of the truth than this. On the other hand, since, as Maarten Maartens puts it, no unmarried woman ever gets over 30, many will remain for a long time at 29. It is doubtful, however, if any woman can remain at that age for 20 years; and anything less than that would not affect our statistics. Shop assistants have a special interest in keeping young, though domestic servants over 25 will run them hard. But, as Miss Collet points out, "ideas as to what constitutes middle age change. The class of persons who, 20 years ago, were afraid of admitting themselves to be 25 years of age, would probably at the present time be content with remaining under 30." Perhaps a time will come when they will own to 40 without faltering, as the progress of society helps us to get over the notion that a woman who has passed her first youth has *ipso facto* forfeited all claim to the pleasures and interests of life.

